# A robust model for read count data in exome sequencing experiments and implications for copy number variant calling

**DOI:** 10.1093/bioinformatics/bts526

**Published:** 2012-08-31

**Authors:** Vincent Plagnol, James Curtis, Michael Epstein, Kin Y. Mok, Emma Stebbings, Sofia Grigoriadou, Nicholas W. Wood, Sophie Hambleton, Siobhan O. Burns, Adrian J. Thrasher, Dinakantha Kumararatne, Rainer Doffinger, Sergey Nejentsev

**Affiliations:** ^1^UCL Genetics Institute, UCL, London, ^2^Department of Medicine, University of Cambridge, Cambridge, ^3^UCL CoMPLEX program, ^4^UCL Institute of Neurology, UCL, ^5^Royal London Hospital, London, ^6^Institute of Cellular Medicine, Newcastle University, Newcastle upon Tyne, ^7^Molecular Immunology Unit, Wolfson Centre for Gene Therapy of Childhood Disease, UCL Institute of Child Health, Great Ormond Street Hospital for Children, London and ^8^Department of Clinical Biochemistry and Immunology, Addenbrookes Hospital, Cambridge, UK

## Abstract

**Motivation**: Exome sequencing has proven to be an effective tool to discover the genetic basis of Mendelian disorders. It is well established that copy number variants (CNVs) contribute to the etiology of these disorders. However, calling CNVs from exome sequence data is challenging. A typical read depth strategy consists of using another sample (or a combination of samples) as a reference to control for the variability at the capture and sequencing steps. However, technical variability between samples complicates the analysis and can create spurious CNV calls.

**Results**: Here**,** we introduce ExomeDepth, a new CNV calling algorithm designed to control for this technical variability. ExomeDepth uses a robust model for the read count data and uses this model to build an optimized reference set in order to maximize the power to detect CNVs. As a result, ExomeDepth is effective across a wider range of exome datasets than the previously existing tools, even for small (e.g. one to two exons) and heterozygous deletions. We used this new approach to analyse exome data from 24 patients with primary immunodeficiencies. Depending on data quality and the exact target region, we find between 170 and 250 exonic CNV calls per sample. Our analysis identified two novel causative deletions in the genes *GATA2* and *DOCK8*.

**Availability:** The code used in this analysis has been implemented into an R package called ExomeDepth and is available at the Comprehensive R Archive Network (CRAN).

**Contact**: v.plagnol@ucl.ac.uk

**Supplementary Information:** Supplementary data are available at *Bioinformatics* online.

## 1 INTRODUCTION

The improvement of DNA sequencing technologies in recent years has radically changed the identification of genetic variants associated with human diseases and in particular, rare disorders (Ng *et al.*, 2010). The use of sequence capture technologies to target protein-coding regions in the human genome followed by high-throughput DNA sequencing (known as exome sequencing) currently provides a cost-efficient approach to discover causal mutations in patients with Mendelian disorders. The majority of published work using exome sequence data focuses on single nucleotide polymorphisms (SNPs) or small insertions/deletions (indels), mostly because short read DNA sequencing technologies are best suited to call these variants. Nevertheless, copy number variants (CNVs), e.g. larger chromosomal indels, also significantly contribute to the aetiology of Mendelian disorders. Three general strategies exist to call CNVs from short read sequence data (Medvedev *et al.*, 2009): split reads (Karakoc *et al.*, 2011; Ye *et al.*, 2009), paired-end reads (Zeitouni *et al.*, 2010) and read depth approaches (Krumm *et al.*, 2012; Sathirapongsasuti *et al.*, 2011; Xie and Tammi, 2009). Read depth analysis is particularly effective for exome data as it does not rely on sequencing into or near the CNV breakpoints. Generally speaking, read depth-based approaches for CNV calling compare the number of reads mapping to a chromosome window with its expectation under a statistical model. Deviations from this expectation are indicative of CNV calls. Similar to the array comparative genomic hybridization (aCGH) methodology, the ratio of read count between a test and a reference sample is usually preferred to a single-sample analysis in order to control for the typically extensive variability in capture efficiency across exons (Krumm *et al.*, 2012; Sathirapongsasuti *et al.*, 2011; Xie and Tammi, 2009). Most of the existing tools for CNV calling that are based on read depth, such as ExomeCNV (Sathirapongsasuti *et al.*, 2011) and CNV-seq (Xie and Tammi, 2009), make Gaussian assumptions about the distribution of read count ratio. In the absence of technical variability, the proportion of reads matching to a specific sample should follow a binomial distribution whose success rate is determined by genome-wide read count ratio between the test sample and the reference set, as well as the potential presence of CNVs. Additional covariates, such as GC content, can alter this success rate in situations where the effects of these covariates vary across samples (Marioni *et al.*, 2007).

Here, we evaluate two different exome sequence datasets and show that Gaussian assumptions generally do not hold. Technical variability at the library preparation, capture and sequencing creates noise that affects the numbers of reads matching to particular exons in a sample-specific manner. As a result, the observed variance exceeds what is predicted by a binomial model that affects the CNV calls. Motivated by this observation, we propose a modified and more robust statistical framework for CNV calling. We apply this model to provide guidelines for the construction of an optimized reference sequence dataset for CNV calling purposes, as well as realistic power estimates. We find that two main factors improve statistical power: increasing the read depth and controlling for any source of technical variability across samples at the capture and sequencing steps. We have developed and coded a new set of tools in an R package called ExomeDepth. We then illustrated its efficiency by discovering novel small causative CNVs in two patients with primary immunodeficiencies, a heterozygous deletion of two exons of the *GATA2* gene and a single-exon homozygous deletion in the *DOCK8* gene.

## 2 SYSTEM AND METHODS

### 2.1 Fitting a robust beta-binomial model for the read depth data

**Fig. 1.**
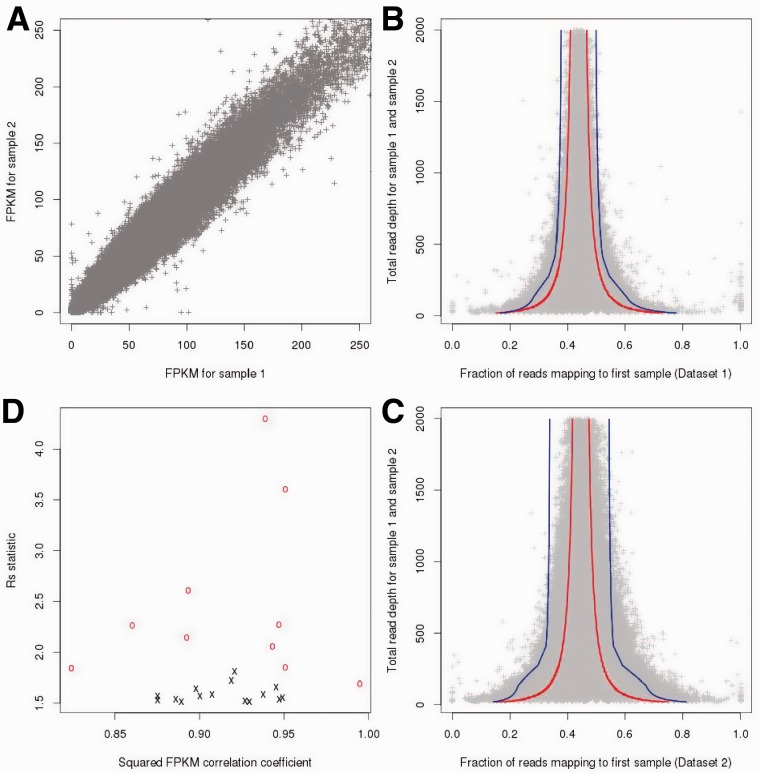
(**A**) Comparison of fragment per kilobase and million base pairs (FPKM) between two exomes (FPKM squared correlation coefficient = 0.992). (**B**) Total read depth for two typical well-matched exomes (*y*-axis) as a function of the proportion of reads mapping to one of two exomes (*x*-axis). The red lines show the 99% confidence interval assuming the best fitting binomial distribution for the read count data. The blue lines show the same 99% confidence interval assuming the best fitting beta-binomial robust model for the same dataset. (**C**) Same as (**B**) but for two typical exomes that are poorly matched to each other. (**D**) 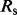 statistic (*x*-axis) and correlation between FPKM values (*y*-axis), both of them computed for each exome with its associated reference set

We analysed the read count data for 24 exome samples from primary immunodeficiency patients (divided into two datasets, Supplementary Table S1 and Section 3). An overview of a normalized measure of read depth [matching fragments per million reads and per kilobase, FPKM, (Mortazavi *et al.*, 2008), Fig. 1A] showed extensive exon–exon variability. Inference of CNV status can therefore, not rely on the highly variable single-sample read count data. However, a comparison between pairs of exome datasets (Fig. 1A) demonstrates the high level of correlations of the normalized read count data across samples (squared FPKM correlation coefficients 0.98–0.988 among 15 exomes in Dataset 1 and 0.72–0.987 for the 9 exomes in Dataset 2). It is therefore possible to use one exome or combine several exomes to construct a reference set to base the CNV inference on. Initially, we analysed pairs of exomes and fitted a binomial model to the genome-wide distribution of read depth data for the reference and the test sample (see Section 3). For the purpose of parameter estimation (but not for subsequent CNV calling steps), we removed exons located in regions harbouring common CNVs (Conrad *et al.*, 2010) to limit the possibility that copy number variable regions increase the variance of the read count ratio. The outcomes of two representative comparisons between a pair of exomes from Dataset 1 and a pair of exomes from Dataset 2 are shown in Figure 1B and C, respectively. The larger variance observed in Figure 1C compared with 1B illustrates that the outcome of this analysis varies extensively, depending on how sequencing and capture were conducted.

The larger variance observed in Figure 1C compared with 1B illustrates that the outcome of this analysis varies extensively depending on how sequencing and capture were conducted. To quantify the variability between B and C, we defined the statistic 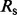 as the ratio between the standard errors of the beta-binomial model and the binomial model (Section 3). This statistic can be intuitively understood as the ratio between the typical distances separating the blue and red curves in Figure 1B and C.

Our results show that a binomial model fails to properly capture the extensive variability in read count ratio across samples. Even in the best case scenario of two well-matched exomes (red line in Fig. 1B), 6.8% of the exons were located outside of the 99% confidence interval. When two exomes were poorly matched (red line in Fig. 1C), a total of 23.2% of exons were outside of the 99% confidence interval. We therefore modified this binomial model and fitted instead a beta-binomial distribution (seeSection 3) to account for the over-dispersion in read count ratio. We further modified the model to account for observed correlations between depth of sequencing and the over-dispersion parameter (Supplementary Fig. S1). This beta-binomial model significantly improved the fit (blue line in Fig. 1B and C). The proportion of exons outside of the 99% confidence interval was reduced to 1.8% for the well-matched pair of exomes and to 2.3% for the poorly matched pair (blue lines in Fig. 1B and C, respectively) To quantify this noise in the sequence data, we defined the statistic 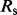 as the ratio between the standard errors of the beta-binomial model and the binomial model. This statistic can be intuitively understood as the ratio between the typical distances separating the blue and red curves in Figure 1B and C. For each sample in Datasets 1 and 2, we estimated the optimum reference set (see below for a description of this procedure) and computed the 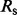 statistic. For Dataset 1 typical values of 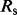 varied between 1.5 and 2, depending on the sample, with an average value of 1.62. For Dataset 2, typical values of 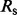 varied between 2 and 4.5 (average: 2.76). The 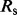 statistic measures the correlations across samples and can be well approximated using the squared pairwise correlation coefficient of FPKM values between the test sample and its associated reference set (Fig. 1D). We hypothesized that some of the differences between samples might be explained by a differential effect of the DNA sequence GC content on capture and sequencing efficiency. Therefore, in the regression analysis, we added GC content as a percentage. The noise reduction was consistent but relatively limited: in Dataset 1, the average 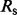 decreased from 1.62 to 1.59. In Dataset 2 the average 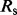 decreased from 2.76 to 2.45.

### 2.2 Power study and optimization of the reference exome set

The different levels of noise illustrated in Figure 1B and C have large implications for the power to detect CNVs. We used a single-exon heterozygous deletion as a typical CNV and estimated the expected value of the posterior probability for this heterozygous deletion given different sets of parameters. We considered three scenarios: 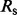 = 1 (absence of any technical bias), 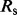 = 1.6 (typical of Dataset 1) and 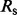 = 2.5 (typical of Dataset 2).

**Fig. 2.**
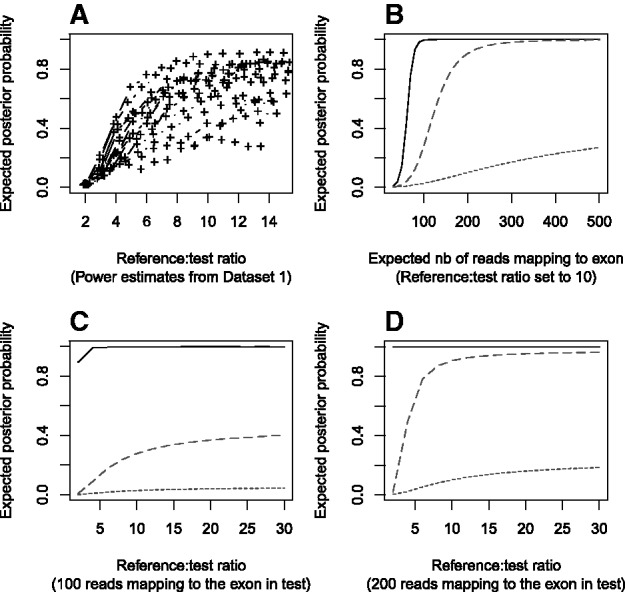
Power study showing the expected posterior probability for a heterozygous deletion call. (**A**) Expected value of the posterior probability (averaged over all exons) for the 15 exomes in Dataset 1 as a function of the (test:reference) read count ratio (which is closely approximated by the number of exomes in the aggregate reference set). Each line shows a different test exome sample and the most correlated exome is added to the reference at each step. (**B**) The expected number of reads that would be mapping to a normal copy number exon varies (along the x-axis) but the (reference:test) sequencing depth remains constant at 10 (i.e. the reference set approximately consists of an aggregate of 10 exomes). Other parameters, including the level of correlations between test and reference exome, are kept constant. Power estimates assume a typical exome from each of the two Datasets 1 and 2 (the median value of the posterior probability is shown). (**C**), (**D**) The number of exomes in the aggregate reference set varies but the expected number of reads mapping to a normal copy number exon for the test sample is set to 100 (C) and 200 (D). For (B), (C) and (D), the black line refers to an optimum dataset in the absence of sample-to-sample technical variability (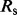 = 1), longer dash to the typical dispersion parameter estimated from Dataset 1 (*R*_s_ = 1.6) and shorter dash for the typical dispersion parameter estimated from Dataset 2 (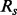 = 2.5)

The construction of the optimum reference set is coded in the select.reference.set function of the ExomeDepth package. To summarize briefly, for each test exome we rank the remaining samples by order of correlation with the test exome. Samples are then added sequentially to the aggregate reference set. At each iteration we fit our robust model and compute the expected value of the posterior probability in favour of a single-exon heterozygous deletion call. This process of adding samples to the aggregate reference stops once the posterior probability stops to increase. This optimization is essentially a trade-off between limiting the variance (by increasing the size of the reference set) and increasing the bias (by adding exome samples to the reference in spite of being less correlated). In Dataset 1 (Fig. 2A) we found that the optimum size of the reference set was ∼10. In several instances adding further samples in the reference set actually decreased the power.

Figure 2B investigates the role of read depth on the power to detect a CNV. For Datasets 1 and 2, we selected the optimum reference sets and extracted the parameters associated with this fit. We investigated the effect of read depth by changing the expected number of reads that map to the heterozygous deleted exon in the test sample. In contrast with Figure 2A, this computation holds the 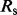 parameter constant, i.e. we assume that all additional exome samples are similarly correlated with the test exome. It therefore only considers the role of read depth and not the added complexity of adding exome samples that are not necessarily as well correlated with the test exome. This analysis showed strong differences between Datasets 1 and 2. In Dataset 1, 300 reads mapping to an exon in the test sample were sufficient to provide complete power, whereas for Dataset 2, the posterior probability in favour of the deletion call could never exceed 30%, even with more than 500 reads. This result indicates that an increase in the read depth cannot compensate for low levels of correlations between the exomes. Using 100-bp paired-end reads, and assuming a 500-bp long exon, 300 mapping reads amount to an average read depth of ∼100. This read depth would need to be twice larger (i.e. 200) if the exon was only 250-bp long. To provide a more general boundary for the size of the reference set, we investigated in Figure 2C and D the behaviour of the power estimates as the size of the reference set increases. As for Figure 2B, we used the optimum parameters estimated in Figure 2A for the median samples in Datasets 1 and 2 and kept the 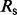 parameter constant. Hence, no bias is created by adding less correlated exome samples and only the effect of the size of the reference set is evaluated. We considered two scenarios of moderate and high read depth (Fig. 2C and D). With these assumptions, while the power keeps increasing, the increase becomes slow once the reference:test ratio reaches a value of 10. This result suggests that very large reference sets would provide only limited increase in power to detect heterozygous deletions. In all tested scenarios, the difference between the power curves estimated from the bias-free model (black line in Fig. 2B–D) compared with the estimates in either of the datasets (red and blue lines) was large. Datasets 1 and 2 also showed substantial difference in power (Fig. 2C and D). These observations illustrate an important effect of variability between individual exomes in a dataset, which is captured by the 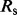 statistic, on the power to detect CNVs. Note that the power to detect single-exon heterozygous deletions in Dataset 2 remains very low and would not reach 1 in any realistic scenario, because the level of noise is too high. Therefore, in Dataset 2 CNVs need to overlap multiple exons to be detectable. Power estimates for heterozygous duplications were much lower than for heterozygous deletions (Supplementary Fig. S2), a difference also commonly observed for all array-based CNV assays. Both datasets lacked power to detect single-exon heterozygous duplications (Supplementary Fig. S2A–D), but larger duplications could be identified. For example, in Dataset 1, and to some extent in Dataset 2, a three-exon heterozygous duplication typically can be detected (Supplementary Fig. S2E–H). Homozygous deletions are naturally easier to detect than heterozygous deletions. Although quantifying the power is complicated by the arbitrary parameterization of the background level of mapping reads, we found that under most realistic assumptions, an expected number of reads greater than 30 mapping to an exon in the test sample was sufficient to identify a homozygous deletion in either of the two datasets.

### 2.3 Characteristics of CNVs and comparison with other tools

The probability for the hidden Markov chain to enter a cn ≠ 2 state sets the sensitivity/specificity balance of ExomeDepth. We parameterized this value using the expected number of CNV calls for an exome sequence (Section 3 and Supplementary Fig. S3). We found the total number of CNV calls to be relatively stable over the range of parameters considered (Supplementary Fig. S3), only increasing sharply for a prior expectation of 1000 CNV calls genome-wide. The ExomeDepth default parameter uses a relatively stringent prior expectation of 20 CNV calls per exome sequence. With this choice, ExomeDepth called a median number of 213 CNVs per sample in Dataset 1, including 62.3% deletions. Consistent with the more limited power in Dataset 2, ExomeDepth identified a lower median number of 177 CNV calls in this dataset (62.9% of them deletions), in spite of a 31.5% larger target region (50 Mb versus 38 Mb). CNVs called by ExomeDepth in Datasets 1 and 2 included a median number of five exons and a median length of 10.6 kb. About 10% of the CNV calls were longer than 100 kb and 0.1% were longer than 500 kb.

Comparison with other algorithms is complicated by the absence of a ‘gold standard’ dataset and the bias inherent to CNV calls in available CNV databases. Nevertheless, the majority of CNVs called in our data should be present in a large-scale database such as the Database of Genomic Variants (DGV), (Zhang *et al.*, 2006). We defined a CNV as previously reported in DGV if a CNV listed in DGV overlaps more than 50% of our CNV call (after excluding DGV CNV calls larger than 500 kb). We found that 13.5% of CNVs in Dataset 1 (20% respectively in Dataset 2) were absent from DGV (Table 1).

**Table 1.** Comparison between our package (ExomeDepth) and two other tools: exomeCopy and ExomeCNV

	exomeDepth	exomeCopy	exomeCNV
**Dataset 1 (*n* = 15)**			
Median nb of CNVs	213	495	2256
Percentage in DGV	86.5	67.8	16.3
Median CNV size (kb)	8.9	1.83	0.16
Median CNV size (exons)	5	3	1
**Dataset 2 (*n* = 9)**			
Median nb of CNVs	177	1228	11 046
Percentage in DGV	80	36.9	26.6
Median CNV size (kb)	12.2	10.04	0.26
Median CNV size (exons)	5	5	1
**1000 Genomes (*n* = 12)**			
Median nb of CNVs	246	641	5261
Percentage in DGV	66	37.2	34.2
Median CNV size (kb)	1.7	9.75	0.34
Median CNV size (exons)	3	4	1
Percentage of known			
CNVs found	75.2	52.8	41.2

We define a CNV called from exome data as ‘in DGV’ (or a ‘known CNV’ in the 1000 Genome analysis) when the CNV in the database overlaps >50% of our CNV call.

To estimate the false negative rate, we used an additional dataset of 12 high-depth exome samples (1000 Genomes Project) for which an independent experiment generated CNV calls using a high-density Nimblegen CGH array [(Conrad *et al.*, 2010) and Section 3]. Combining all 12 samples, the aCGH experiment identified 1344 exonic CNV calls (303 unique calls). Conrad *et al.* (2010) estimate that 40% of CNVs can be genotyped with their experimental design that translates into an approximate expected number of 280 exonic CNV calls per sample, which is broadly consistent with our findings.

To compare our algorithm with existing tools, we first tested ExomeCNV (Sathirapongsasuti *et al.*, 2011). Its underlying model assumes that the distribution of read count ratio between the test and reference exome is Gaussian and a similar assumption is made by CNV-Seq (Xie and Tammi, 2009). Second, we tested exomeCopy (Love *et al.*, 2011), which uses a negative binomial model that is related to our beta-binomial approach. In each case, we followed the methods suggested by these publications and we used the suggested default parameters. Venn diagrams summarizing the overlap between calling algorithms are shown in Supplementary Figure S4.

Comparison between these three tools using a dataset of 12 exomes from 1000 Genomes and the two datasets from this study highlighted a clear trend. First, ExomeDepth is more conservative than the other tools, with the median number of CNV calls between 177 and 246 per exome, whereas exomeCopy and ExomeCNV called numerous additional CNVs (Table 1 and Supplementary Fig. S4). Second, ExomeDepth detected 75.2% of the known exonic CNVs in the 12 exome samples from the 1000 Genomes Project (CNVs identified by the independent aCGH experiment). This was markedly higher than the fraction of CNVs identified by exomeCopy (52.8%) and ExomeCNV (41.2%; Table 1), indicating a higher sensitivity. Interestingly, the difference between our analysis and exomeCopy was more limited for Dataset 1, for which the exomes are better matched to each other, than for Dataset 2 and the 1000 Genomes dataset, consistent with the fact that the aggregate reference optimization step implemented in ExomeDepth is more helpful when the variability across samples is larger.

### 2.4 Discovery of two novel and likely disease-causing deletions in the *GATA2* and *DOCK8* genes in patients with primary immunodeficiency

**Fig. 3.**
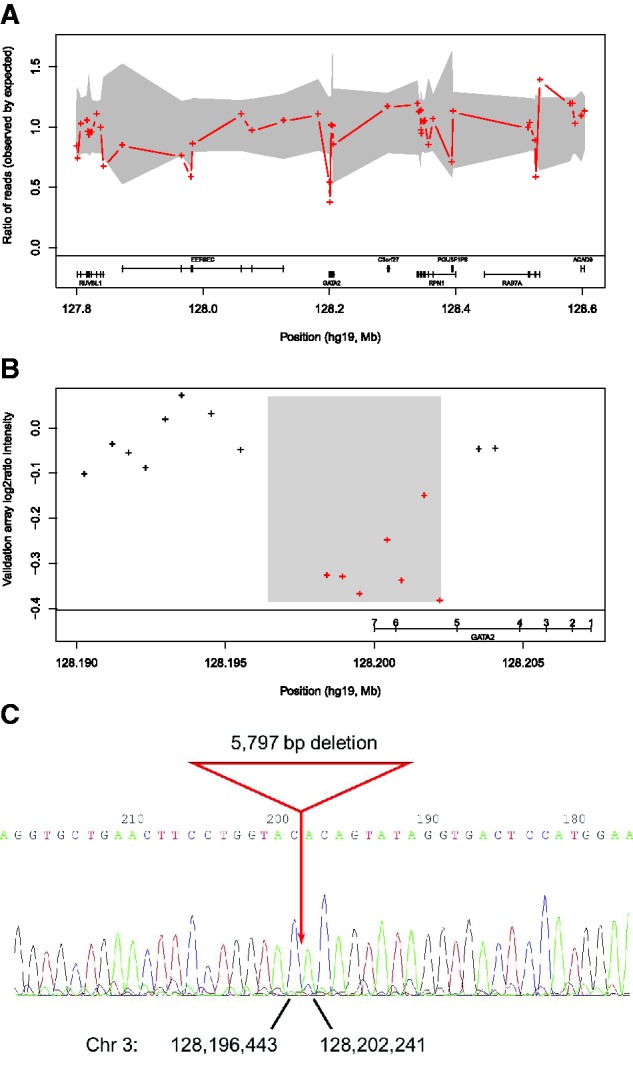
(**A**) Heterozygous deletion of exons 6 and 7 of the *GATA2* gene identified by ExomeDepth in the exome sequence data. The red crosses show the ratio of observed/expected number of reads for the test sample. The grey shaded region shows the estimated 99% confidence interval for this observed ratio in the absence of CNV call. The presence of two contiguous exons with read count ratio located outside of the condfidence interval is indicative of a heterozygous deletion in this sample. Independently, each exon would yield a posterior probability for the deletion call of 15% (exon 6) and 77% (exon 7). The combined CNV call has a posterior probability >99.9%. (**B**) Validation of a 6-kb deletion using a targeted array CGH (Agilent 15K format) containing 26 probes in the *GATA2* gene region. Each cross indicates a probe and red crosses indicate probes located in the region of a heterozygous deletion. (**C**) Sequencing of the deletion breakpoints identified the exact boundaries of this 5797-bp deletion overlapping exons 6 and 7 of the *GATA2* gene

We then investigated if any of the newly discovered rare CNVs in our data affects genes that previously have been involved in primary immunodeficiencies. In patient P1 ExomeDepth identified a heterozygous deletion of the consecutive exons 6 and 7 of the *GATA2* gene with a read count ratio <0.5% quantile (Fig. 3A). Independently, each exon would yield a posterior probability for the deletion call of 77% (exon 7) and 15% (exon 6). The combined CNV call has a posterior probability >99.9%. We then designed a custom CGH array (Supplementary Data) containing 26 probes in the *GATA2* gene region. In this patient we validated a heterozygous deletion of 6 kb that included *GATA2* exons 6 and 7 (Fig. 3B). We then amplified the breakpoint region, sequenced it and mapped the exact boundaries of this 5797-bp deletion (Fig. 3C; coordinates chr3: 128, 196, 444-128, 202, 240). The clinical presentation for this patient was consistent with previous reports of heterozygous variants in the *GATA2* gene (Ostergaard *et al.*, 2011), indicating that this two exons deletion is very likely to be causal for P1 (Supplementary Data for clinical details).

In another patient (P2), ExomeDepth identified a deletion of a single exon 8 of the *DOCK8* gene (Supplementary Fig. S5A). This CNV call had a posterior probability >99.99%. Complete absence of reads mapping to exon 8 is indicative of a homozygous deletion. We sequenced the deletion breakpoints and identified the exact boundaries of a 3197-bp deletion (Supplementary Fig. S5B; coordinates chr9: 323, 591–326, 787). The clinical presentation is consistent with previous reports involving homozygous mutations in the *DOCK8* (Zhang *et al.*, 2009) indicating that this variant is almost certainly causal (Supplementary Data for clinical details).

## 3 IMPLEMENTATION

### 3.1 Primary immunodeficiency patients

We investigated 24 patients who suffered from severe and/or disseminated recurrent infections, and have been diagnosed with primary immunodeficiencies (PIDs). Of these patients, 13 were of European descent and 11 patients were of Asian descent, of which, 5 originated from consanguineous families. All material from patients was obtained with informed consent from adults and from the parents of children who participated in the study in accordance with the Declaration of Helsinki and with approval from the ethics committees (04/Q0501/119, amendment 2; 06/Q0508/16 and 10/H0906/22).

### 3.2 Exome data

We isolated DNA samples from blood or peripheral blood mononuclear cells (PBMCs). Exome sequence data have been generated in two batches. Dataset 1 comprises exomes from 15 patients and Dataset 2 comprises exomes from 9 patients. For exome target enrichment Agilent SureSelect 38 Mb and 50 Mb kits have been used for samples in Datasets 1 and 2, respectively. Samples in both datasets have been sequenced using Illumina HiSeq with 94-bp paired-end reads. Reads were aligned to the hg19 reference sequence using the software novoalign (www.novocraft.com). Single sample summary statistics are provided in Supplementary Table S1.

### 3.3 Read count computation

Exon locations were defined using the Ensemble database release version 57 (human genome build hg19). We only considered autosomal chromosomes (chr1–22) to avoid the additional complication of gender (in which case, male and female samples would need to be analysed separately). Analysis of the GC content for each exon uses the same Ensembl release and the Perl Ensembl API tool. We used the R package Rsamtools to extract the read count information from the individual BAM files. All reads were paired-end. We only included consistent paired reads (i.e. both reads located <1000 bp away from each other and in the correct orientation) and with Phred scaled mapping quality ≥20. The location was defined by the middle location between the extreme ends of both paired reads. Exons closer than 50 bp were merged into a single location owing to the inability to properly separate reads mapping to either of them. After merging close exons, we considered a total number of 229 056 autosomal exons.

### 3.4 Statistical model

We denote the exonic read count X for the test sample and Y for the aggregate reference. Assuming that the distribution of the read count ratio 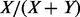 is only determined by the relative read depth of the test and reference samples, an appropriate model is binomial with the probability that a random read is assigned to the test sample is:
(1)
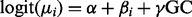

where 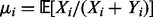 is the probability that a random read belongs to the test sample (rather than the reference). The intercept parameter 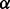 is estimated separately for each test sample. GC refers to the GC content. The index *i* denotes the exon and the covariate 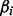 relates to the copy number status for the exon *i*: the proportion of reads mapping to the test sample for deletions/duplications is computed based on the expected proportion for normal copy number and assuming a read ratio of 0.5 (for a deletion) or 1.5 (for a duplication). A motivation for our work is the fact that this binomial model does not fully capture sample specific biases. We propose instead the robust beta-binomial model (Agresti, 2002):
(2)
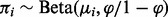

(3)
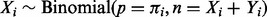

where the over-dispersion parameter 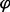 is numerically estimated from the read count data. Assuming this model, the mean value of the beta binomial variable *X* remains unchanged but its variance becomes 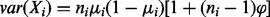 where 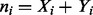, adding to the binomial variance an additional over-dispersion term. Last, the addition of GC content to the model contributes to predicting individual specific biases.

However, an analysis of the data showed that a single 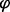 typically could not fully summarize the read count variance over the full range of read depth (Supplementary Fig. S1). We therefore, modified the model to allow the parameter 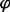 to take different values depending on the total read count. We used a linear extrapolation to combine these estimates over the full range of read depth. The number of intervals for the read count data is set to two by default and can be modified by the user. Supplementary Figure S1 describes this fitting process in more details.

### 3.5 Numerical estimation

We fitted the binomial logistic model described in Equation (1) using the glm function in R. We fitted the beta-binomial model described in Equations (2) and (3), including the maximum likelihood estimation of the over-dispersion parameter 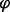, using a maximum likelihood approach implemented in the R package aod. The procedure estimates for each exon, an expected read ratio 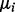 and a genome-wide over-dispersion parameter 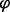. This parameter estimation is done assuming cn = 2 for all exons. In a second step, and for each exon, covariates for deletions/duplications are added to estimate the likelihood of the read count data for the scenarios cn = 1 and 3. In the beta-binomial models expressed in Equations (2) and (3), the beta-binomial distribution is usually parameterized using two parameters *a* and *b* [mean 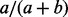 and variance 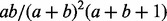]. The regression formulation of the model described above links to this distribution with 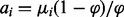 and 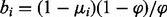. Prior to fitting these models (Fig. 1B and C), we removed exons located in regions harbouring common CNVs (Conrad *et al.*, 2010) to limit the possibility that such CNVs significantly increase the variance of the read count ratio.

### 3.6 Hidden Markov chain and choice of prior probabilities

For each exon, our beta-binomial model generates a likelihood value under three distinct scenarios (copy number = deletion, normal, duplication). To combine the likelihood across multiple exons we used a hidden Markov model. Each step of the hidden Markov state corresponds to one exon in the human genome. This model serves the double purpose of merging CNV calls across exons, as well as specifying a prior probability of observing a CNV for each exon. This prior probability is coded into the transition probability of the hidden Markov chain between the normal copy number state (cn = 2) and either of the copy number variable states (cn = 1 or cn = 3). We parameterized this before using the expected number ne of CNVs *a-priori*, i.e. the probability of transitioning from cn = 2 to cn = 1 or cn = 3 is 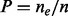 where *n* = 229 056 is the total number of exons. We set the default model such that, from the hidden state cn = 2, the probability to move into a deletion state is the same as the probability to move into a duplication state. In a deletion/duplication state, the underlying Markov chain has a default probability 0.5 to revert back to cn = 2 and a probability 0.5 to remain in the same deletion/duplication state. To provide a set of calls for each sample, we use the maximum likelihood Viterbi algorithm. Each version of our statistical models in Equations (1–3) generates a likelihood under the cn = 1, 2, 3 scenarios. For CNV with lower (cn = 0 for homozygous deletion) or higher (cn 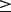 4) number of DNA copies, the model with cn = 1, 2 and 3 hidden rejects the null with added confidence compared with the simpler scenarios cn = 1 or cn = 3. Hence, we found no benefit in considering additional copy number states (besides, cn = 1, 2 and 3). Rather, we estimate copy number state using the read count ratio after the CNV is detected. Importantly, copy number is always estimated with respect to the reference and the absolute value cannot be estimated by this procedure.

### 3.7 Power estimates

Owing to the common particular interest in discovering loss-of-function variants we considered a heterozygous deletion as a typical scenario for our power estimations (Fig. 2). The heterozygous duplication case is considered in Supplementary Figure S2. Following the parameter estimation step, we can generate for each CNV, and given the test and reference read count data X and Y, a Bayes factor BF = 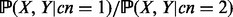. Using our selected prior distribution (*P* = 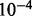 of observing a CNV, i.e. corresponding to an expected number of 20 CNV calls genome-wide), we compute the expected value of the posterior distribution for the CNV call.

### 3.8 Choice of samples for the comparative analysis

We downloaded from the 1000 Genomes a dataset of 12 high-depth exome samples generated using the Solid sequencing technology (single-end reads), for which an independent experiment generated CNV calls using a high-density Nimblegen CGH array (Conrad *et al.*, 2010). These 12 samples are NA18502, NA19099, NA19239, NA19240 (Yoruba) and NA06985, NA1199, NA11995, NA12004, NA12044, NA12156, NA12414, NA12489 (CEPH).

### 3.9 Parameters of ExomeCNV/exomeCopy in the comparative analysis

To apply ExomeCNV we followed the instructions provided by the user guide, using 0.9999 as threshold for sensitivity and specificity, with the software set to optimize the specificity. For each test sample (labelled tumour sample in the ExomeCNV analysis), the aggregegate reference set (labelled as normal) consisted of the remaining exomes from the same dataset. We then applied the classify.eCNV function with default parameters setting the admixture rate to 0 (because we are not concerned by a mixture with tumour DNA). Finally, multi.CNV.analyze was used to obtain the final list of merged calls from the list of exonic CNV calls. For exomeCopy the read count data was estimated using the R built-in functions provided by this package. For each sample, the background noise was estimated on the basis of GC content and the read count data from the other exomes in the same dataset. We fitted the negative-binomial model and the hidden Markov chain using the steps recommended in the package vignette and the default parameters. For the exomeCopy analysis, the recommendation to split long exons into smaller units (and therefore, get more uniform numbers of reads in each bin) increased the number of CNV calls and the concordance with DGV dropped, strongly suggesting that this step did not improve the overall accuracy. We therefore used our set of exon delimited regions to compute the read count.

## 4 DISCUSSION

We have developed a novel CNV calling methodology using read depth information from exome sequence data and implemented it within an R package called ExomeDepth. This allowed us to maximize the statistical power to detect even small CNVs in the presence of technical variability inherent to high-throughput DNA sequencing technologies. As a consequence, compared with other tools, we found the greatest improvement for datasets that show more technical variability across samples. Although ExomeDepth is designed to be used as a standalone software, the contruction of a reference set is a problem shared across all read depth CNV calling algorithms for exome data. Therefore, additional refinements proposed by other CNV calling algorithms to improve calling accuracy could potentially be applied after ExomeDepth has identified the optimum aggregate reference set.

Because ExomeDepth assumes that the CNV call is not present in the reference sample, it is best suited to call rare CNVs. Nevertheless, our analysis of exome samples from the 1000 Genome Project indicates that it can call common CNVs as well, even though some power is lost when the allele frequency is high. Our computations indicate that the power to detect rare CNVs is maximized for a reference:test ratio of ∼10:1. Hence, while we find no obvious benefit in using a very large dataset (> 100 exomes), it is essential to generate exome data in batches of six or more samples.

Our analysis shows that a Gaussian model for the read count data is not appropriate for exome sequence data, which is likely to explain the discrepancy with the observed larger number of CNV calls generated by ExomeCNV. The discrepancy with exomeCopy is more surprising, because exomeCopy fits a robust negative binomial model related to our model. Additionally, in the exomeCopy analysis, we used the optimized reference set identified by the ExomeDepth analysis. The default parameters of the exomeCopy hidden Markov chain were also similar. Comparing both methods, we find that the main difference is that exomeCopy essentially takes a profile likelihood approach: it uses the median normalized read depth at each exon to account for the variability in exon capture efficiency, which is a nuisance parameter. However, the median read depth can be unreliable, if the sample size is small and/or the data are noisier (e.g. in Dataset 2). Instead, in ExomeDepth we used a logistic model, which deals with that nuisance parameter by conditioning on the total read count for each exon. We hypothesize that exomeCopy would show results more similar to ours, if the exon-specific parameters were estimated within the negative binomial model rather than prior to model fitting. However, the number of exons, and therefore, the number of parameters to estimate, would be large which makes it difficult in practice.

In our study the statistical power to detect CNVs varied extensively between two datasets and was largely determined by sample-to-sample variability within datasets that emerged either at the exome capture or at the sequencing step. This feature of the data cannot be detected by commonly used single-sample quality metrics: it is the correlations across samples rather than the single-sample summary statistic that are relevant. Owing to its essential role for CNV calling, we argue that a measure of sample-to-sample consistency (e.g. correlation between FPKM values) should be provided by sequencing facilities when exomes are analysed in sufficiently large batches.

Finding of the *GATA2* and *DOCK8* deletions illustrates the power of ExomeDepth to identify even heterozygous and small CNVs comprising just one to two exons. We conclude that reducing technical variability between the samples and using bioinformatics tools that maximize statistical power of CNV detection, such as ExomeDepth, will allow efficient CNV identification and will increase the value of the future exome sequencing experiments.

## Supplementary Material

Supplementary Data

## References

[bts526-B1] Agresti A (2002). Categorical data analysis. Wiley Series in Probability and Statistics.

[bts526-B2] Conrad DF (2010). Origins and functional impact of copy number variation in the human genome. Nature.

[bts526-B3] Karakoc E (2011). Detection of structural variants and indels within exome data. Nat. Methods.

[bts526-B4] Krumm N (2012). Copy number variation detection and genotyping from exome sequence data. Genome Res..

[bts526-B5] Love MI (2011). Modeling read counts for CNV detection in exome sequencing data. Stat. Appl. Genet. Mol. Biol..

[bts526-B6] Marioni JC (2007). Breaking the waves: improved detection of copy number variation from microarray-based comparative genomic hybridization. Genome Biol..

[bts526-B7] Medvedev P (2009). Computational methods for discovering structural variation with next-generation sequencing. Nat. Methods.

[bts526-B8] Mortazavi A (2008). Mapping and quantifying mammalian transcriptomes by RNA-Seq. Nat. Methods.

[bts526-B9] Ng SB (2010). Exome sequencing identifies the cause of a mendelian disorder. Nat. Genet..

[bts526-B10] Ostergaard P (2011). Mutations in GATA2 cause primary lymphedema associated with a predisposition to acute myeloid leukemia (Emberger syndrome). Nat. Genet..

[bts526-B11] Sathirapongsasuti JF (2011). Exome sequencing-based copy-number variation and loss of heterozygosity detection: ExomeCNV. Bioinformatics.

[bts526-B12] Xie C, Tammi MT (2009). CNV-seq, a new method to detect copy number variation using high-throughput sequencing. BMC Bioinformatics.

[bts526-B13] Ye K (2009). Pindel: a pattern growth approach to detect break points of large deletions and medium sized insertions from paired-end short reads. Bioinformatics.

[bts526-B14] Zeitouni B (2010). SVDetect: a tool to identify genomic structural variations from paired-end and mate-pair sequencing data. Bioinformatics.

[bts526-B15] Zhang J (2006). Development of bioinformatics resources for display and analysis of copy number and other structural variants in the human genome. Cytogenet. Genome Res..

[bts526-B16] Zhang Q (2009). Combined immunodeficiency associated with DOCK8 mutations. New Engl. J. Med..

