# Supplementary material for: A robust model for read count data in exome sequencing experiments and implications for copy number variant calling
Source: Bioinformatics. 2012 Aug 31;28(21):2747–54. doi: 10.1093/bioinformatics/bts526 (PMC3476336; doi:10.1093/bioinformatics/bts526)
Supplement: Supplementary Data [file supp_28_21_2747__index.html]

A robust model for read count data in exome sequencing experiments and implications for copy number variant calling — A robust model for read count data in exome sequencing experiments and implications for copy number variant calling — Supplementary Data 

# A robust model for read count data in exome sequencing experiments and implications for copy number variant calling

## Supplementary Data

files

**Files in this Data Supplement:**

- Supplementary Data - pdf file
