# Supplementary material for: A robust model for read count data in exome sequencing experiments and implications for copy number variant calling
Source: Bioinformatics. 2012 Aug 31;28(21):2747–54. doi: 10.1093/bioinformatics/bts526 (PMC3476336; doi:10.1093/bioinformatics/bts526)
Supplement: Supplementary Data [file supp_bts526_supplemental.pdf]

Supplemental information- A robust model for read count data in exome sequencing experiments and implications for copy number variant calling

Vincent Plagnol[1], James Curtis[2], Michael Epstein[1,3], Kin Mok[4], Emma Stebbings[2], Sofia Grigoriadou[5], Nicholas W. Wood[4], Sophie Hambleton[6], Siobhan O. Burns[7], Adrian Thrasher[7], Dinakantha Kumararatne[8], Rainer Doffinger[8] and Sergey Nejentsev[2]

	Sample ID	Target	Percent of (unique) reads aligned	Mean exonic depth	Fraction of exons with depth > 10	nreads	Nb of reads (PCR dups excluded)	Dataset
1	dataset1_sample1	Agilent 38 Mb	0.91	53.71	0.83	43292170	41421592	1
2	dataset1_sample2	Agilent 38 Mb	0.90	67.29	0.85	57690737	54200765	1
3	dataset1_sample3	Agilent 38 Mb	0.90	67.08	0.82	57360072	54256598	1
4	dataset1_sample4	Agilent 38 Mb	0.90	57.40	0.83	47431645	44882549	1
5	dataset1_sample5	Agilent 38 Mb	0.90	64.24	0.84	55695762	52048638	1
6	dataset1_sample6	Agilent 38 Mb	0.91	64.23	0.85	51772970	47503998	1
7	dataset1_sample7	Agilent 38 Mb	0.90	62.03	0.86	52130874	47957133	1
8	dataset1_sample8	Agilent 38 Mb	0.91	69.74	0.87	56156472	52221559	1
9	dataset1_sample9	Agilent 38 Mb	0.90	61.13	0.84	50226018	46822985	1
10	dataset1_sample10	Agilent 38 Mb	0.91	62.60	0.84	50997014	48080000	1
11	dataset1_sample11	Agilent 38 Mb	0.91	64.66	0.84	54954565	51470849	1
12	dataset1_sample12	Agilent 38 Mb	0.90	57.10	0.84	46372387	43113661	1
13	dataset1_sample13	Agilent 38 Mb	0.91	59.20	0.85	47542320	44481106	1
14	dataset1_sample14	Agilent 38 Mb	0.91	56.55	0.84	49955448	45645148	1
15	dataset1_sample15	Agilent 38 Mb	0.90	60.85	0.84	52316350	47871136	1
16	dataset2_sample1	Agilent 50 Mb	0.87	39.26	0.81	60146855	56345325	2
17	dataset2_sample2	Agilent 50 Mb	0.89	81.25	0.90	129869404	124080578	2
18	dataset2_sample3	Agilent 50 Mb	0.85	43.19	0.77	64894230	59926702	2
19	dataset2_sample4	Agilent 50 Mb	0.87	44.33	0.78	65210339	62484907	2
20	dataset2_sample5	Agilent 50 Mb	0.87	40.54	0.76	61399721	57889353	2
21	dataset2_sample6	Agilent 50 Mb	0.88	57.67	0.84	82264872	77893321	2
22	dataset2_sample7	Agilent 50 Mb	0.86	44.96	0.76	61311414	54894252	2
23	dataset2_sample8	Agilent 50 Mb	0.88	37.34	0.76	57055948	52313738	2
24	dataset2_sample9	Agilent 50 Mb	0.88	66.53	0.88	108676909	103666247	2

Supplemental S 1: Summary statistics for the 24 exomes from primary immune deficiency patients used in this study.

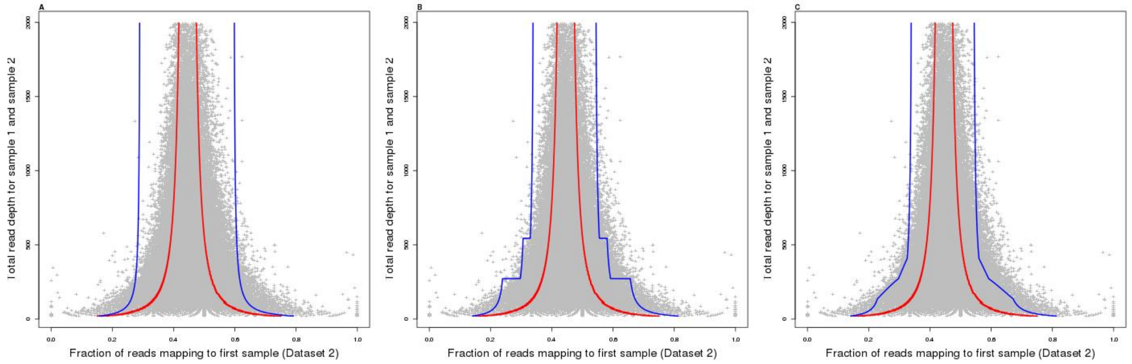

Supplemental S 1: Model fitting for the over-dispersion parameter ϕ. For graphs A, B and C, the red lines show the 99% confidence interval after fitting a binomial distribution to the data. For A, B and C, the blue lines show different versions of the beta-binomial model. In A, a single value of ϕ is used to fit the read count data. In graph B, three different values of ϕ are used depending on read depth. In graph C, the same three values are used but a linear interpolation is used to obtain continous values of ϕ across the full range of read depth.

Estimation for the over-dispersion parameter ϕ

We observed that while a standard beta-binomial distribution improved greatly the fit to the data, the estimated value of the over-dispersion ϕ should be different across different ranges of read depth. We therefore divided the exon data in discrete bins, and in the same estimation procedure different values of ϕ were estimated for each bin. An example of this model fitting is shown in Figure S1.

Design of the CGH array to map the *GATA2* deletion

To validate and refine the breakpoints of the deletion in the GATA2 region we used a custom designed Agilent 15K comparative genomic hybridization array. DNA samples were sent to and processed by Oxford Gene Technology. The array included approximately equally spaced probes, 26 probes in a 24 kb region (chr3:128,180,104-128,204,118) defined around the GATA2 gene (on average one probe every 0.92 kb).

Sequencing of the *GATA2* and *DOCK8* breakpoints

To PCR the regions containing novel deletions we used the following primers: for GATA2
5cagcactcatgacagcacct3 and 5tctctttcccgaatcccttt3;
for DOCK8
5AATGTCCCAAGGAACACCTG3 and 5AGATTGGGCAAATCAGATCG3; We then used Sanger sequencing with multiple internal primers to identify the exact location of breakpoints.

Details on clinical symptoms for patients P1 (*GATA2*) and P2 (*DOCK8*)

Patient P1 is a female of the Arabic descent who presented with fever at the age of 23 years. Mycobacterium avium intracellulare has been isolated from her bronchoalveolar lavage fluid and lymph node biopsy material. Monocytopenia, B and NK cell deficiency have been found in the peripheral blood and myelodysplastic syndrome has been diagnosed on bone marrow examination. Karyotype analysis revealed monosomy 7 mosaicism in the patients blood cells. One of the patients eleven siblings has been also diagnosed with myelodysplastic syndrome and then developed acute myeloid leukemia and another sibling died at the age of 7 months due to an unknown cause. Recently, heterozygous mutations of the *GATA2* gene have been reported in several clinically related conditions. These include sporadic monocytopenia and mycobacterial infection (MonoMAC) syndrome (Hsu *et al.*, 2011), an immunodeficiency syndrome characterised by the loss of dendritic cells, monocytes, B and NK cells (DCML deficiency) Dickinson *et al.* (2011), myelodysplastic

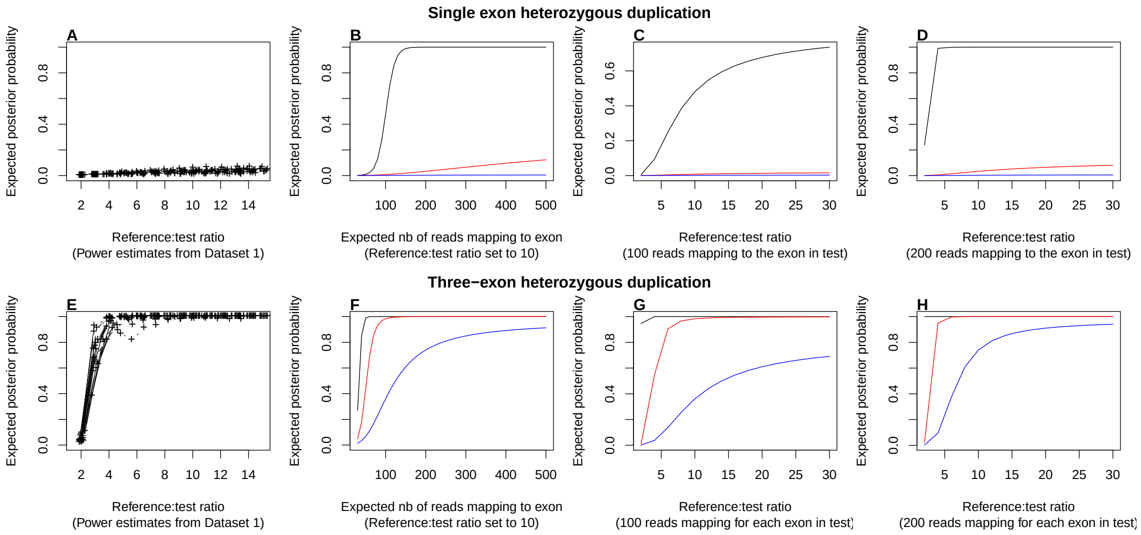

Supplemental S 2: Expected value of the posterior probability for a single exon heterozygous duplication (A, B, C and D) and a three-exon heterozygous duplication (E, F, G and H). For B, C, D and F, G, H, the black line refers to an optimum dataset in the absence of sample-to-sample technical variability ($R_s = 1$), red to the typical dispersion parameter estimated from Dataset 1 ($R_s = 1.6$) and blue for the typical dispersion parameter estimated from Dataset 2 ($R_s = 2.5$).
A, E: Expected value of the posterior probability (averaged over all exons) for the 15 exomes in Dataset 1 as a function of the depth of the reference sequence. Each line represents one exome sample and each cross a different choice for the reference sample.
B, F: The expected number of reads that would be mapping to a normal copy number exon varies (x axis) but the (reference:test) sequencing depth remains constant at 10. Other parameters, including the level of correlations between test and reference exome, are kept constant. Power estimates assume a typical exome from Dataset 1 and 2 (selected based on median value of the posterior).
C, D, G, H: The (reference:test) sequencing depth ratio varies (x-axis) but the expected number of reads mapping to a normal copy number exon for the test sample is set to 100 (C, G) and 200 (D, H).

syndrome and acute myeloid leukemia (Hahn *et al.*, 2011), and the Emberger syndrome Ostergaard *et al.* (2011). Many of the reported patients have mutations that affected zinc finger 2 domain encoded by exons 6 and 7 of the GATA2 gene Hyde and Liu (2011). Given these similarities in clinical presentation and genetic defect, we concluded that the disease in patient P1 has been caused by a heterozygous deletion in the GATA2 gene.

Patient P2 was born to consanguineous parents and suffered from infantile eczema with varicella-associated flare, cows milk protein intolerance, lymphadenitis in the second year of life, two episodes of Streptococcus pneumoniae septicaemia (one associated with septic arthritis) and chronic carriage of non-typhoidal salmonella. Laboratory evaluation showed elevated IgE, intermittent lymphopenia and pronounced eosinophilia. Homozygous mutations in the *DOCK8* gene have been reported in patients that suffered from recurrent infections, such as streptococcal pneumonia and cutaneous infections caused by herpesviruses, including varicella-zoster virus. Most of the reported patients had severe atopy, increased IgE levels and eosinophilia (Zhang *et al.*, 2009; Engelhardt *et al.*, 2009). These similarities in clinical presentation and genetic mutations indicate that homozygous deletion of exon 8 in the *DOCK8* gene is causative in patient P2.

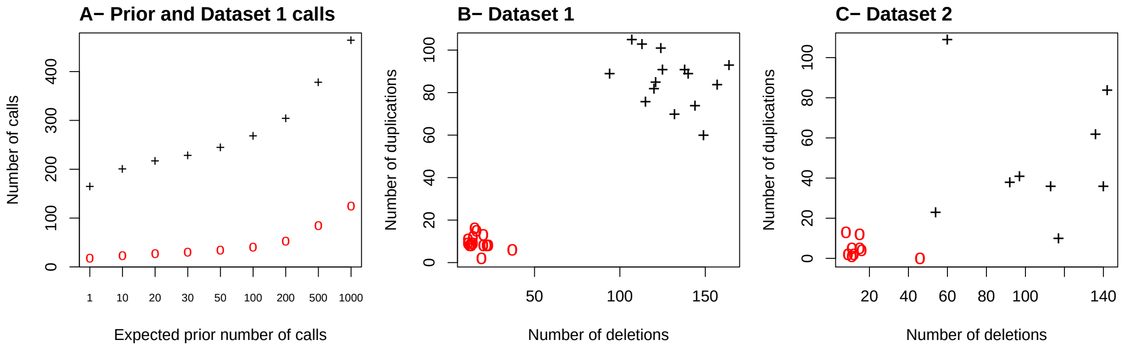

Supplemental S 3: A-Average number of CNVs per sample (black crosses: overall, red circles: absent from the Database of Genomic Variants) as a function of the expected number of calls under the prior distribution. B- Dataset 1: Number of deletions (x-axis) and duplications (y-axis) assuming a prior expected number of CNV calls of 20. Each point corresponds to one exome sample (black crosses: total CNV number, red circles: CNVs absent from the Database of Genomic Variants).
C: Same as B but for Dataset 2.

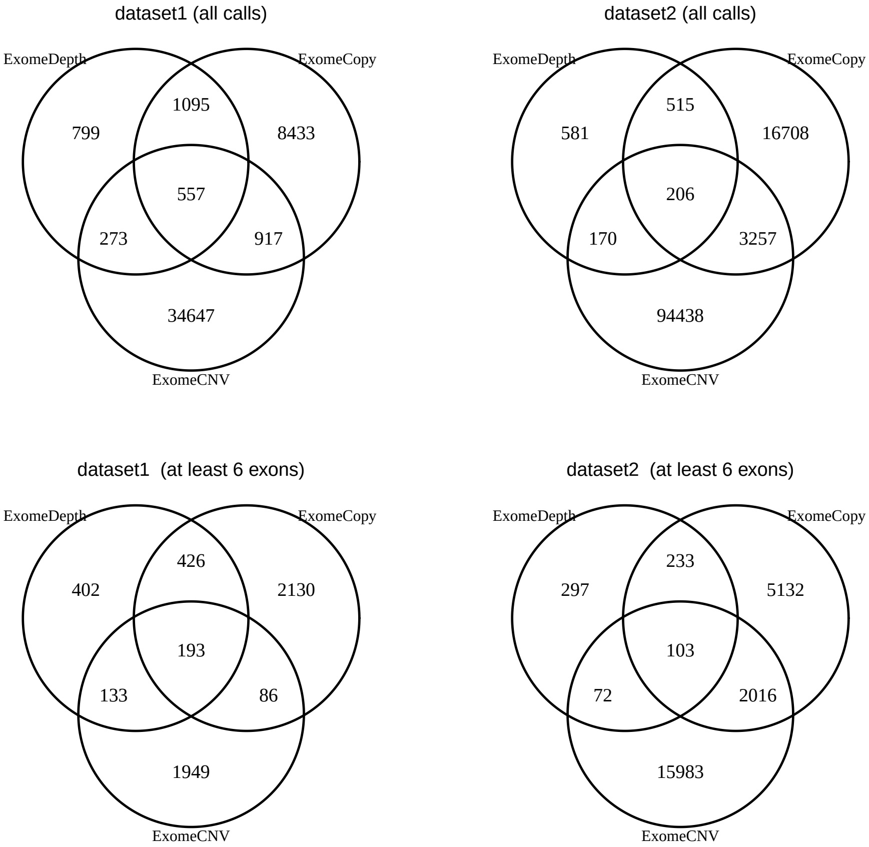

Supplemental S 4: Venn diagrams describing the overlap between the three calling algorithms tested in this study (ExomeDepth, exomeCopy and ExomeCNV) for dataset 1 (left) and dataset 2 (right). We considered either all calls (top row) or only CNV calls that include 7 or more exons (bottom row). A call was defined as shared if the reciprocal overlap between CNV calls was at least 25% of the overall length of each call.

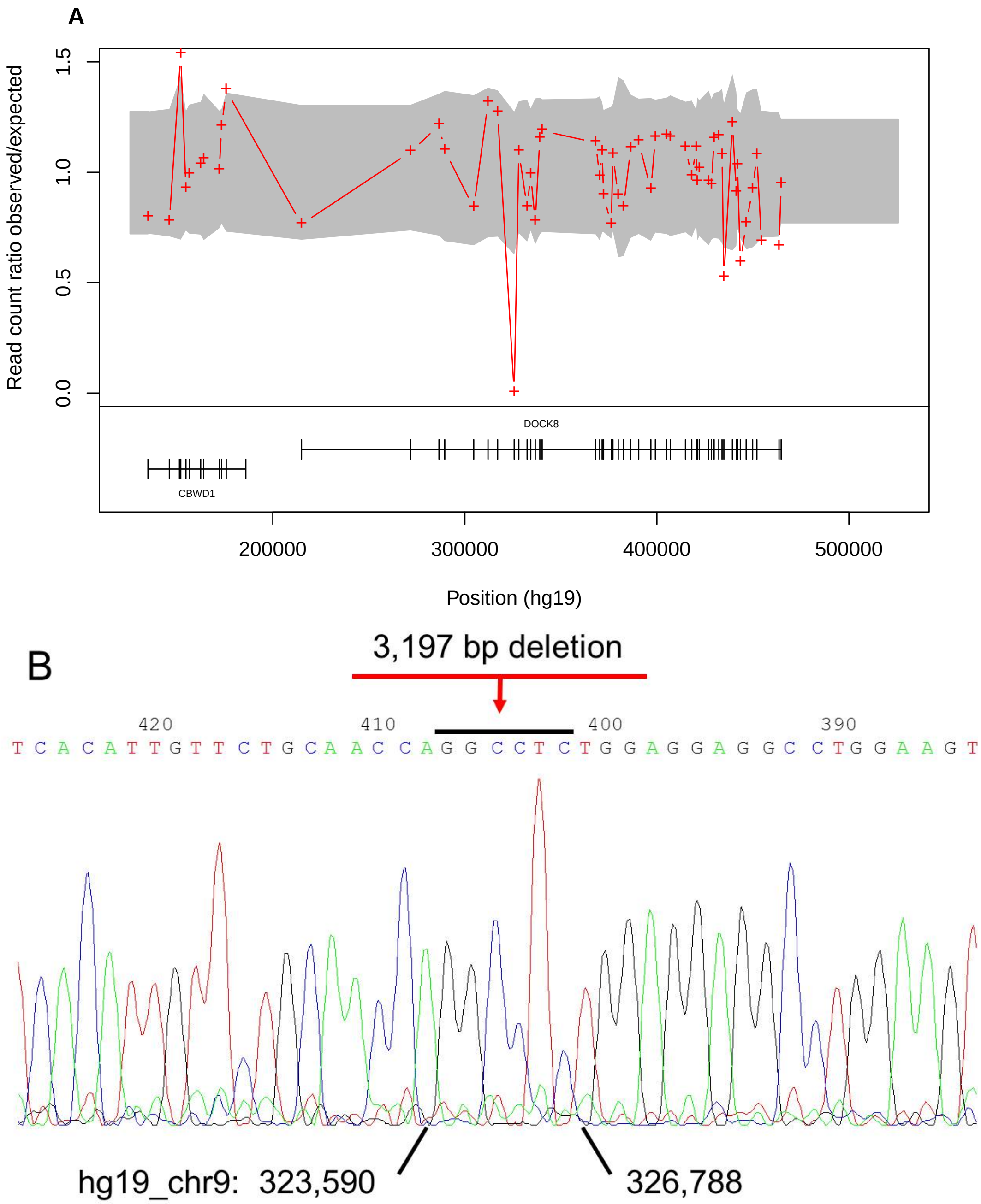

Supplemental S 5: A: Homozygous deletion of exon 8 of the *DOCK8* gene identified by ExomeDepth in the exome sequence data. The red crosses show the ratio of observed/expected number of reads for the test sample. The grey shaded region shows the estimated 99% condence interval for this observed ratio in the absence of CNV call. The CNV call has a posterior probability of greater than 99.99%. B: Sequencing of the deletion breakpoints identified the exact boundaries of a 3,197-bp deletion overlapping exon 8 of the *DOCK8* gene. The deletion breakpoints are located in the introns flanking exon 8 of *DOCK8*. In this patient the deleted region is replaced by six nucleotides, GGCCTC.

References

Dickinson, R. E., Griffin, H., Bigley, V., Reynard, L. N., Hussain, R., Haniffa, M., Lakey, J. H., Rahman, T., Wang, X.-N., McGovern, N., Pagan, S., Cookson, S., McDonald, D., Chua, I., Wallis, J., Cant, A., Wright, M., Keavney, B., Chinnery, P. F., Loughlin, J., Hambleton, S., Santibanez-Koref, M., and Collin, M. (2011). Exome sequencing identifies GATA-2 mutation as the cause of dendritic cell, monocyte, B and NK lymphoid deficiency. *Blood*, pages blood–2011–06–360313–.

Engelhardt, K. R., McGhee, S., Winkler, S., Sassi, A., Woellner, C., Lopez-Herrera, G., Chen, A., Kim, H. S., Lloret, M. G., Schulze, I., Ehl, S., Thiel, J., Pfeifer, D., Veelken, H., Niehues, T., Siepermann, K., Weinspach, S., Reisli, I., Keles, S., Genel, F., Kutukculer, N., Kutuculer, N., Camciolu, Y., Somer, A., Karakoc-Aydiner, E., Barlan, I., Gennery, A., Metin, A., Degerliyurt, A., Pietrogrande, M. C., Yeganeh, M., Baz, Z., Al-Tamemi, S., Klein, C., Puck, J. M., Holland, S. M., McCabe, E. R. B., Grimbacher, B., and Chatila, T. A. (2009). Large deletions and point mutations involving the dedicator of cytokinesis 8 (DOCK8) in the autosomal-recessive form of hyper-IgE syndrome. *The Journal of allergy and clinical immunology*, **124**(6), 1289–302.e4.

Hahn, C. N., Chong, C.-E., Carmichael, C. L., Wilkins, E. J., Brautigan, P. J., Li, X.-C., Babic, M., Lin, M., Carmagnac, A., Lee, Y. K., Kok, C. H., Gagliardi, L., Friend, K. L., Ekert, P. G., Butcher, C. M., Brown, A. L., Lewis, I. D., To, L. B., Timms, A. E., Storek, J., Moore, S., Altree, M., Escher, R., Bardy, P. G., Suthers, G. K., D'Andrea, R. J., Horwitz, M. S., and Scott, H. S. (2011). Heritable GATA2 mutations associated with familial myelodysplastic syndrome and acute myeloid leukemia. *Nature genetics*, **43**(10), 1012–1017.

Hsu, A. P., Sampaio, E. P., Khan, J., Calvo, K. R., Lemieux, J. E., Patel, S. Y., Frucht, D. M., Vinh, D. C., Auth, R. D., Freeman, A. F., Olivier, K. N., Uzel, G., Zerbe, C. S., Spalding, C., Pittaluga, S., Raffeld, M., Kuhns, D. B., Ding, L., Paulson, M. L., Marciano, B. E., Gea-Banacloche, J. C., Orange, J. S., Cuellar-Rodriguez, J., Hickstein, D. D., and Holland, S. M. (2011). Mutations in GATA2 are associated with the autosomal dominant and sporadic monocytopenia and mycobacterial infection (MonoMAC) syndrome. *Blood*, **118**(10), 2653–5.

Hyde, R. K. and Liu, P. P. (2011). GATA2 mutations lead to MDS and AML. *Nature Genetics*, **43**(10), 926–927.

Ostergaard, P., Simpson, M. A., Connell, F. C., Steward, C. G., Brice, G., Woollard, W. J., Dafou, D., Kilo, T., Smithson, S., Lunt, P., Murday, V. A., Hodgson, S., Keenan, R., Pilz, D. T., Martinez-Corral, I., Makinen, T., Mortimer, P. S., Jeffery, S., Trembath, R. C., and Mansour, S. (2011). Mutations in GATA2 cause primary lymphedema associated with a predisposition to acute myeloid leukemia (Emberger syndrome). *Nature genetics*, **43**(10), 929–931.

Zhang, Q., Davis, J. C., Lamborn, I. T., Freeman, A. F., Jing, H., Favreau, A. J., Matthews, H. F., Davis, J., Turner, M. L., Uzel, G., Holland, S. M., and Su, H. C. (2009). Combined immunodeficiency associated with DOCK8 mutations. *The New England journal of medicine*, **361**(21), 2046–55.
